# Successful implantation of cardiac resynchronization therapy in a patient with coronary sinus reducer using proximal coronary sinus branches: a case report

**DOI:** 10.1093/ehjcr/ytae662

**Published:** 2024-12-11

**Authors:** Jacopo Costantino, Lorenzo Maria Zuccaro, Barbara Romani, Francesco Luigi Rotolo, Daniele Porcelli

**Affiliations:** Department of Clinical Sciences of Anesthesiology and Cardiovascular Sciences, Sapienza University of Rome, Piazzale Aldo Moro, 00162 Rome, Italy; Cardiac Electrophysiology and Electrostimulation Unit, San Pietro Fatebenefratelli Hospital Rome, Via Cassia, 600, 00189 Rome, Italy; Department of Medicine, University of Padua, Via Giustiniani, 2, 35128 Padua, Italy; Cardiac Electrophysiology and Electrostimulation Unit, San Pietro Fatebenefratelli Hospital Rome, Via Cassia, 600, 00189 Rome, Italy; Cardiac Electrophysiology and Electrostimulation Unit, San Pietro Fatebenefratelli Hospital Rome, Via Cassia, 600, 00189 Rome, Italy; Cardiac Electrophysiology and Electrostimulation Unit, San Pietro Fatebenefratelli Hospital Rome, Via Cassia, 600, 00189 Rome, Italy; Cardiac Electrophysiology and Electrostimulation Unit, San Pietro Fatebenefratelli Hospital Rome, Via Cassia, 600, 00189 Rome, Italy

**Keywords:** Case report, Coronary sinus reducer, Cardiac resynchronization therapy, Chronic coronary syndrome, Heart failure

## Abstract

**Background:**

The coronary sinus reducer (CSR) is a therapeutic option for patients with coronary artery disease who are not eligible for further revascularization and experience refractory angina. Cardiac resynchronization therapy (CRT) improves symptoms and prognosis in heart failure with reduced ejection fraction, but the presence of a CSR may complicate left ventricular lead placement. Only four cases have been reported so far in this context. This case report introduces a novel technique for left ventricular lead implantation in such patients.

**Case summary:**

A 73-year-old man with a history of chronic coronary syndrome, coronary artery bypass surgery, angioplasties, and CSR implantation presented with heart failure symptoms. His ECG showed sinus rhythm and left bundle branch block (QRS 160 ms), and echocardiography revealed severe systolic dysfunction (ejection fraction 20%). During Cardiac Resynchronization Therapy-Defibrillator (CRT-D) implantation, venography revealed a suitable proximal tributary branch near the CSR, which was successfully used for lead placement without complications.

**Discussion:**

The CSR has shown promise in relieving refractory angina. As ischaemic heart disease progresses, CRT may become necessary in patients with CSR. The CSR’s design can reduce the vascular lumen and complicate lead placement. Previous reports have confirmed the technical feasibility of CRT with CSR but lack long-term safety data. This case highlights that, under favourable anatomical conditions, proximal tributaries of the coronary sinus can be used for lead placement, potentially avoiding complications from reduced venous outflow.

**Learning points**

- It is possible to implant a cardiac resynchronization therapy even in patients with coronary sinus reducer.
- During the decision-making process, it is important to perform coronary sinus venography to assess the presence of collateral branches proximal to the coronary sinus region before ruling out this therapy *a priori*.

## Introduction

In patients with coronary artery disease who are not candidates for further revascularization and have angina refractory to medical therapy, the implantation of cardiac resynchronization therapy (CRT) has recently emerged as an effective treatment option.^1^ Ischaemic heart disease is the leading cause of heart failure with reduced ejection fraction; in this context, CRT has proven to be effective in improving symptoms and prognosis.^2^ It remains uncertain whether the presence of the reducer constitutes a significant impediment to left ventricular lead placement. To date, only four cases have been described of CRT implantation in this specific circumstance.^3,4^ This case report describes a novel left ventricular catheter implantation technique that has not been previously reported.

## Summary figure

**Figure:**
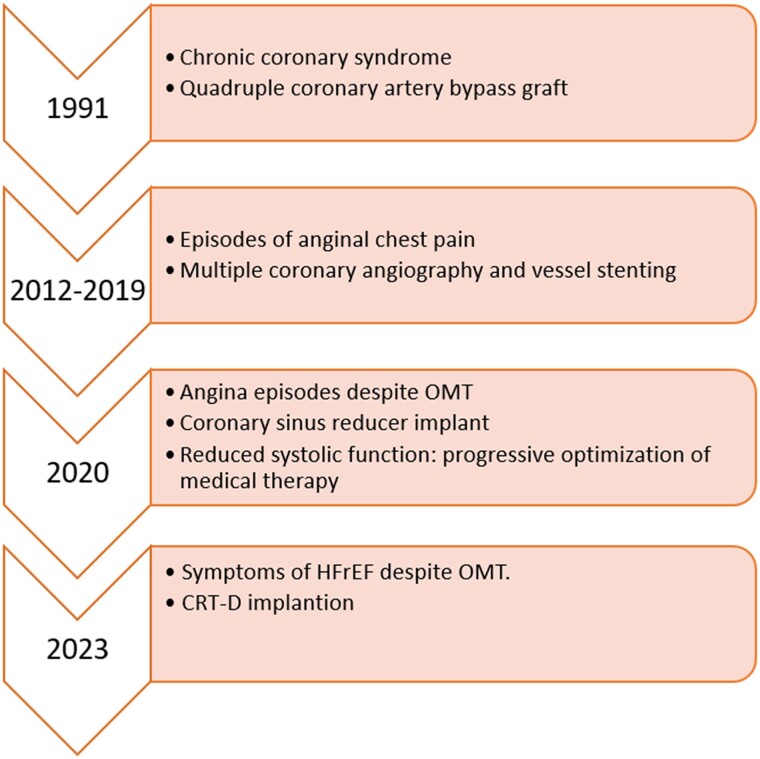


## Case presentation

A 73-year-old man with a history of chronic coronary syndrome who underwent quadruple coronary artery bypass grafting 29 years earlier, along with subsequent multiple angioplasty procedures, was referred for coronary sinus reducer (CSR) implantation 3 years ago (Neovasc Inc.) for angina refractory to medical therapy (CCS class III) and coronary artery disease not amenable to further revascularization. He presented again with symptoms of heart failure (NYHA class III) despite optimized medical therapy (including bisoprolol 5 mg o.d., sacubitril/valsartan 49/51 mg b.i.d., potassium canrenoate 50 mg o.d., dapagliflozin 10 mg o.d., furosemide 250 mg b.i.d., ranolazine 750 mg b.i.d., acetylsalicylic acid 100 mg o.d., rosuvastatin 40 mg o.d., and ezetimibe 10 mg o.d.). The ECG showed sinus rhythm at 70 b.p.m., left bundle branch block (QRS 160 ms), and the echocardiogram revealed a dilated left ventricle with severe impairment of systolic function (ejection fraction 20%), without evidence of significant valvular disease. There was therefore an indication for CRT with a defibrillator.

**Figure 1:**
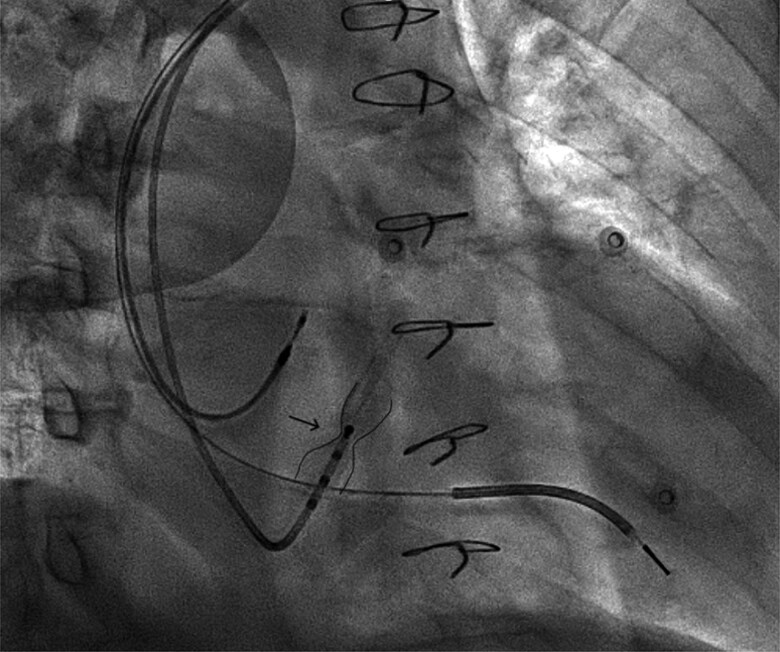
CS (coronary sinus) cannulation using a 5 Fr electrophysiology catheter. The CSR (coronary sinus reducer) is highlighted. RAO (right anterior oblique) angiographic projection.

**Figure 2:**
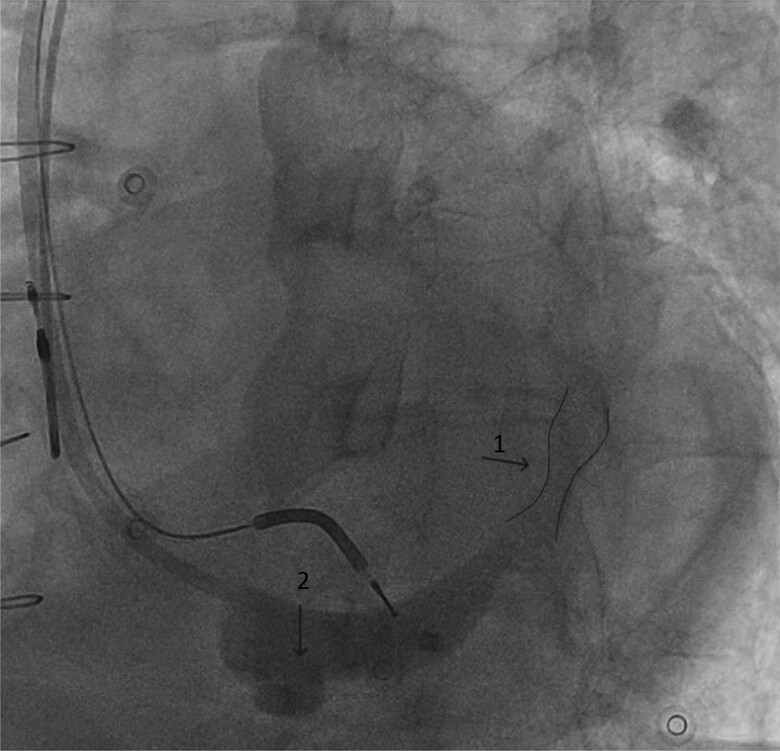
CS venography. Note the CRS (arrow 1), the tributary branch of the CS (arrow 2). The CSR is highlighted. AP (anteroposterior) angiographic projection.

**Figure 3:**
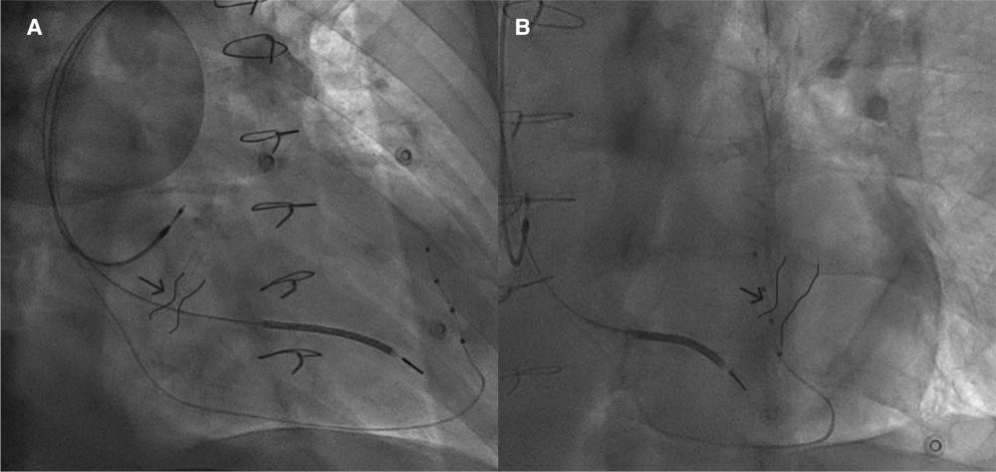
Final position of the catheter for left ventricular stimulation in a tributary branch of the CS. The CSR indicated by the arrow is highlighted. In panel (*A*), RAO angiographic projection. In panel (*B*), LAO (left anterior oblique) angiographic projection.

**Figure 4:**
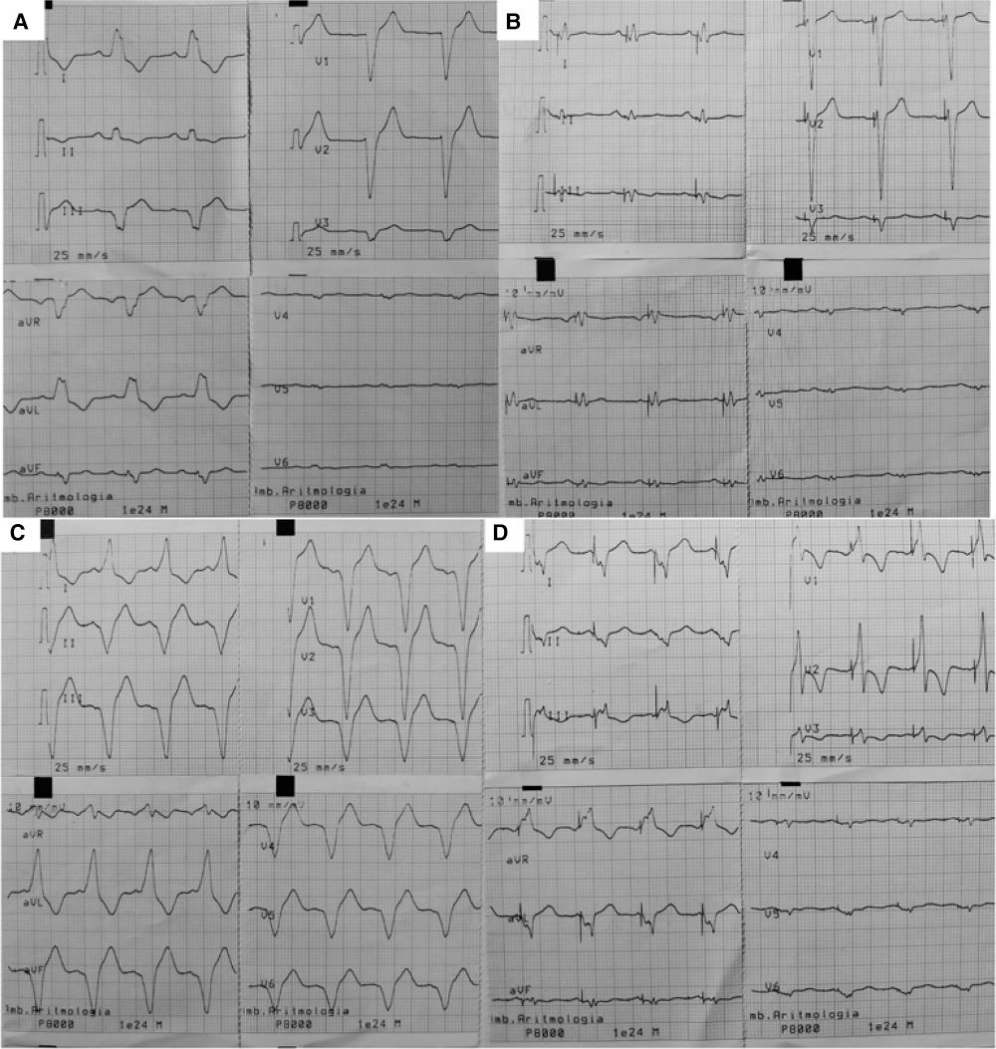
Spontaneous and pacing electrocardiograms. Panel (*A*) shows the spontaneous ECG tracing, panel (*B*) illustrates the ECG tracing during biventricular pacing, panel (*C*) illustrates the ECG tracing during right ventricular pacing, and panel (*D*) illustrates the ECG tracing during the left ventricular pacing.

To cannulate the coronary sinus, a 5 Fr electrophysiology catheter was successfully advanced through the reducer while the 9 F delivery sheath was positioned proximal to the reducer (*Figure 1*). A venography of the coronary sinus was performed, which showed the CSR with a reduction in the calibre of the vascular lumen and the presence of a tributary branch of the coronary sinus proximal to the CSR (*Figure 2*). An angiographic guide was then advanced to branch of the coronary sinus previously identified proximal to the reducer, and the left ventricular catheter (ACUITY™ X4 quadripolar 5 F; Boston Scientific) was positioned using an over-the-wire approach without complications (*Figure 3*). The electrical parameters were satisfactory, and the QRS duration showed a significant reduction (*Figure 4*). Three months later, we observed significant clinical improvement, including enhanced functional capacity, a reduction in oedema, a marked decrease in NT-proBNP levels, and a substantial reduction in the need for furosemide.

## Discussion

Although the experience in using CSR is limited, the emerging data on its effectiveness in improving the symptoms of patients suffering from refractory angina are promising.^1^ In fact, the implantation of CSR has been considered by the 2024 Chronic Coronary Syndrome Guidelines Task Force; in this ESC document, the use of CSR is recommended (class IIb, level of evidence B) to alleviate the disabling symptoms of refractory angina in patients with optimized medical therapy and coronary artery disease not amenable to further revascularization.^5^ The exact pathophysiological mechanism by which this device reduces angina symptoms is not yet fully understood. The currently most accepted hypotheses are primarily two: the redistribution of blood from the less ischaemic subepicardium to the more ischaemic subendocardium, and the process of neoangiogenesis.^6^

Since many patients with ischaemic heart disease may develop left ventricular dysfunction over time, CRT implantation may subsequently be required in some CSR recipients. However, this system may present certain technical difficulties. The CSR is designed as an expandable, hourglass-shaped stainless steel stent, with the diameter of its central portion being ∼9 F (3 mm). The quadripolar catheter for left ventricular stimulation has a diameter of 5 F, which makes its positioning inside the CSR not only challenging but may also cause a significant reduction in the vascular lumen, potentially leading to reduced venous outflow and increased venous pressure in the coronary sinus. Although previously published case reports indicate that it is technically possible to position the left ventricular catheter through the CSR,^3,4^ there are currently no data on the safety of this procedure, potential complications (e.g. coronary sinus obstruction and coronary sinus thrombosis), or possible long-term haemodynamic consequences. Our case report, however, demonstrates how it is possible, through venography, to identify collateral tributaries of the coronary sinus proximal to the CSR implantation. If the anatomy is favourable, these tributaries can potentially be used for the correct positioning of the left ventricular lead without the need to navigate through the CSR. We recognize the concern that not targeting the postero-lateral vein could potentially affect QRS narrowing, which is important for achieving optimal outcomes. Nonetheless, we believe that this approach may still be effective and could offer some safety benefits by minimizing the risk of unexpected long-term complications associated with excessive reduction of venous outflow. Conversely, if the anatomy is not favourable, it may be advisable to consider stimulation of the conduction system; recent studies have suggested that this can be an effective and safe alternative to conventional biventricular pacing, although the current evidence is still limited.^7^

## Conclusions

This case report demonstrates that CRT implantation is a feasible treatment option even in patients with CSR. Unlike the previously described cases, this report shows that, in patients with favourable anatomy, it is possible to successfully implant the left ventricular catheter by identifying and utilizing tributaries of the coronary sinus proximal to the CSR, thus avoiding the need to navigate within it.

## Data Availability

Additional datasets used and/or analysed during the current study are available from the corresponding author on reasonable request.

## Lead author biography

Dr Jacopo Costantino earned his medical degree from La Sapienza University of Rome, Italy. He is currently a Cardiology Fellow at La Sapienza University of Rome and a PhD Candidate at the University of Padua. His primary research and clinical interests focus on electrophysiology and cardiomyopathies.
